# Chronic *Trichuris muris* Infection Decreases Diversity of the Intestinal Microbiota and Concomitantly Increases the Abundance of Lactobacilli

**DOI:** 10.1371/journal.pone.0125495

**Published:** 2015-05-05

**Authors:** Jacob Bak Holm, Daniel Sorobetea, Pia Kiilerich, Yuliaxis Ramayo-Caldas, Jordi Estellé, Tao Ma, Lise Madsen, Karsten Kristiansen, Marcus Svensson-Frej

**Affiliations:** 1 Laboratory of Genomics and Molecular Biomedicine, Department of Biology, University of Copenhagen, Copenhagen, Denmark; 2 Immunology Section, Department of Experimental Medical Sciences, Medical Faculty, Lund University, Lund, Sweden; 3 INRA, UMR1313 Génétique Animale et Biologie Intégrative (GABI), Jouy-en-Josas, France; 4 National Institute of Nutrition and Seafood Research, Bergen, Norway; Virginia Tech University, UNITED STATES

## Abstract

The intestinal microbiota is vital for shaping the local intestinal environment as well as host immunity and metabolism. At the same time, epidemiological and experimental evidence suggest an important role for parasitic worm infections in maintaining the inflammatory and regulatory balance of the immune system. In line with this, the prevalence of persistent worm infections is inversely correlated with the incidence of immune-associated diseases, prompting the use of controlled parasite infections for therapeutic purposes. Despite this, the impact of parasite infection on the intestinal microbiota, as well as potential downstream effects on the immune system, remain largely unknown. We have assessed the influence of chronic infection with the large-intestinal nematode *Trichuris muris*, a close relative of the human pathogen *Trichuris trichiura*, on the composition of the murine intestinal microbiota by 16S ribosomal-RNA gene-based sequencing. Our results demonstrate that persistent *T*. *muris* infection dramatically affects the large-intestinal microbiota, most notably with a drop in the diversity of bacterial communities, as well as a marked increase in the relative abundance of the *Lactobacillus* genus. In parallel, chronic *T*. *muris* infection resulted in a significant shift in the balance between regulatory and inflammatory T cells in the intestinal adaptive immune system, in favour of inflammatory cells. Together, these data demonstrate that chronic parasite infection strongly influences the intestinal microbiota and the adaptive immune system. Our results illustrate the complex interactions between these factors in the intestinal tract, and contribute to furthering the understanding of this interplay, which is of crucial importance considering that 500 million people globally are suffering from these infections and their potential use for therapeutic purposes.

## Introduction

The gastrointestinal tract harbours a myriad of microorganisms, collectively termed the microbiota, which have evolved complex interdependent relationships with the host[1, 2]. The microbiota is critical to the health of the host by metabolising nutrients[3, 4], maturing the immune system[5–7] and competing out pathogens[8, 9], as evidenced by studies utilising mice raised under germ free conditions devoid of commensal organisms. Thus, resident bacteria shape the host immune system and small disturbances in the balance between different microbial communities can have adverse effects on its integrity. Likewise, activation of the immune system, for example during infection, may lead to alterations in the composition of the microbiota[10, 11], and thereby affect the balance of the intestinal microenvironment. An imbalance in this host-microbial relationship is thought to contribute to multiple inflammatory and autoimmune diseases[6, 12].

Parasitic worms are among the most prevalent pathogens that afflict humans, and they share a long evolutionary history with us. Hence, worm infections are rarely lethal but can nonetheless cause a wide variety of health issues such as abdominal pain, anaemia, stunted growth and impaired cognitive development[13, 14]. On the other hand, parasites may incur benefits to the host by educating the immune system early in life and providing signals that serve to dampen inflammation and strengthen the regulatory immune response[15]. Signs of this may be seen in developed countries, where the absence of worm infections has been correlated with an increased incidence of various immune-associated diseases including allergies, inflammatory bowel disease (IBD), multiple sclerosis, rheumatoid arthritis (RA) and type 1 diabetes (T1D)[16–18]. Encouraged by results from various laboratory models[19–24], much emphasis has been placed on the potential therapeutic value of parasitic infections in treating human diseases[25–28]. Indeed, therapeutic infection with the porcine nematode *Trichuris suis* is currently in clinical trial for treatment of IBD[29]. Interestingly, diseases that have been associated with the absence of parasitic worms, including IBD, RA and T1D, have also been correlated with alterations in the intestinal microbiota[30]. It is therefore plausible that the lack of parasitic worm infections, via modulation of the intestinal microbiota, might confer protection against these disorders.

*Trichuris muris* is the murine-specific counterpart to the human pathogen *Trichuris trichiura* that infects approximately 500 million people globally[31]. Its life cycle follows a strict faecal-oral route. Thus, following infection of the large intestine, *T*. *muris* worms remain in the caecal and colonic epithelium throughout their lifespan. During the first three weeks after hatching, the larvae reside embedded in syncytial tunnels formed through adjacent intestinal epithelial cells, but from the L2-L3 moulting stage, worms start protruding out into the intestinal lumen, reaching adulthood five weeks after infection. In line with other intestinal parasites, protective immunity to *T*. *muris* infection is dependent on induction of a T helper cell type 2 (Th2) response[32]. However, at sufficiently low doses, *T*. *muris* establishes a chronic infection by invoking a Th1 response characterised by the production of the cytokine interferon-γ (IFN-γ)[33].

Given that *T*. *muris* larvae occupy the same niche as the majority of the intestinal microbiota it is plausible that these organisms influence each other, which may subsequently affect the intestinal microenvironment of the host. Interestingly, oral administration of *Lactobacillus casei* can increase the susceptibility to *T*. *muris* infections[34] and intestinal luminal bacteria are important for enabling the *T*. *muris* larvae to hatch during the initial infection[35], further illustrating the complex inter-species relationships that characterise the intestinal microenvironment. However, data on the influence of *Trichuris* parasites on intestinal bacterial communities is limited. Studies in pigs infected with *T*. *suis* have demonstrated changes in bacterial diversity and metabolic networks after infection[36, 37], whereas studies in humans infected with *T*. *trichiura* have yielded conflicting data[38, 39], emphasising the need for further research.

We set out to study the effect of chronic *T*. *muris* infection on the gut microbiota and immune response. Here, we provide evidence that persistent *T*. *muris* infection resulted in dramatic alterations in microbial communities, in both the caecum and colon, which became apparent approximately three weeks after infection and became increasingly established with time. Overall, these changes resulted in a less diverse microbiota, and were characterised by a marked increase in the relative abundance of the bacterial family *Lactobacillaceae*. In addition, chronic *T*. *muris* infection affected the balance between inflammatory and regulatory immune cells in the intestinal mucosa, although this seemed to occur prior to the increase in *Lactobacillaceae*. These findings highlight the importance of understanding the intricate interactions of the microbiota, particularly with regard to parasitic worm infections, and their contribution to health and disease.

## Materials and Methods

### Mice

Mice were obtained from Harlan Laboratories (An Venray, Netherlands). Experiments were conducted with age-matched, male C57BL/6 mice that were eight weeks old at the start of the experimentation. Mice were sacrificed by cervical dislocation.

### Ethics Statement

All experiments were conducted in strict accordance with animal welfare laws, as determined by Swedish authorities (Swedish Board of Agriculture, Act 1988:534). The protocol was approved by Malmö/Lund Ethical Board for Animal Research, Lund/Malmö, Sweden (permit no. M467-12), and all efforts were made to minimize suffering of the mice. Mice were monitored daily for signs of stress or disease, such as condition of fur and general movement. None of the mice developed diarrhoea or other intestinal-related issues.

### Trichuris muris

*T*. *muris* (strain E) was maintained, and worm-derived excretory/secretory (E/S) antigens were generated and purified as previously described[40]. To obtain a chronic *T*. *muris* infection, mice were infected with a low dose of approximately twenty infective eggs in sterile-filtered (0.2 μm) tap water by oral gavage. To assess the worm burden of infected mice, large intestines were excised and frozen at -20°C. During the analysis, intestines were cut longitudinally, and scraped free of worms, which were subsequently counted under a reverse phase-contrast microscope.

### Experimental Outline

Mice were co-housed for at least two weeks prior to experimentation to ensure normalisation of their microbiota, and were subsequently placed in individual cages during experiments to avoid cross-contamination. Experiments were conducted according to the scheme in S1 Fig 10 mice were infected at day 0 with a low dose *T*. *muris* eggs and 20 mice were left uninfected. 10 of the uninfected were sacrificed at day 0 while the remaining 10 were sampled over a 35 days period alongside with the 10 infected mice. Fresh faeces were sampled regularly throughout the experiment, after each larval moulting stage: L2 (day 13), L3 (day 20) and L4 (day 27). The faecal samples were immediately frozen on dry ice upon collection. At the end point (day 35), luminal contents were collected from both the caecum and colon. We sampled the luminal colon content distally, to make them correspond to the fresh faeces samples. For simplicity, luminal colon content sampled upon termination and fresh faeces sampled throughout the infection are collectively referred to as “faecal samples”. Finally, to ensure that mice had been properly infected, a separate group of five mice was infected and sacrificed at day 35 for assessment of their worm burden.

### Cell Isolation

Mesenteric lymph nodes (MLN) were stripped of surrounding adipose tissue and mashed in Dulbecco’s phosphate-buffered saline (DPBS; Life Technologies), followed by filtration through 70 μm cell strainers (Fisher Scientific). The large intestines were stripped of attached adipose tissue, opened longitudinally and washed thoroughly in DPBS to remove the faeces. To isolate cells from the large-intestinal lamina propria (LI LP) the intestines were cut into approximately one cm pieces, and incubated thrice in epithelial dissociation buffer consisting of Hank’s balanced salt solution (HBSS; Life Technologies) supplemented with 15 mM 4-(2-hydroxyethyl)-1-piperazineethanesulfonic acid (HEPES; Life Technologies), 2% foetal bovine serum (FBS; Sigma-Aldrich), 5 mM ethylenediaminetetraacetic acid (EDTA; Merck-Millipore), 100 U/ml penicillin + 100 μg/ml streptomycin (Life Technologies), 50 μg/ml gentamicin (Life Technologies) and 1.25 μg/ml Fungizone (Life Technologies) for 15 minutes at 37°C, on continuous shaking. For the first round of treatment, 1 mM DL-dithiothreitol (DTT; Sigma-Aldrich) was added to aid the removal of mucous. After a brief wash, the remaining tissue pieces were subsequently enzymatically digested in R10 buffer consisting of RPMI 1640 (Life Technologies) supplemented with 10 mM HEPES, 10% FBS, 2 mM L-glutamine (Life Technologies), 1 mM sodium pyruvate (Life Technologies), 100 U/ml penicillin + 100 μg/ml streptomycin, 50 μg/ml gentamicin and 1.25 μg/ml Fungizone, along with 0.3 Wünsch-units/ml liberase TM (Roche), 30 μg/ml DNase I (Roche) and 5 mM CaCl_2_ for 45 minutes at 37°C with magnetic stirring. The resulting cell suspension was filtered through 100 μm cell strainers (Fisher Scientific), and subjected to a density gradient centrifugation using Percoll (GE Healthcare) according to manufacturer’s instructions. Briefly, cells were suspended in 40% Percoll and centrifuged over a 70% Percoll layer for 20 minutes, 600 g without brake at room temperature. Cells were collected from the 40/70 interphase and washed with R10 buffer. Cell numbers were assessed with a KX-21N automated hematology analyzer (Sysmex).

### *Ex vivo* Cell Stimulations and Cytokine Analyses

For cytokine secretion analyses, cells were suspended in R10 buffer, seeded at 2.5 x 10^6^ cells/ml in TC MicroWell 96U Nunclon plates (Thermo Fisher Scientific), and incubated with 50 μg/ml E/S antigens for 48 hours at 37°C, 5% CO_2_. Cell-free supernatants were collected and frozen at -20°C for subsequent analyses. Cytokine secretion was measured with BD cytometric bead array (BD Biosciences) according to manufacturer’s instructions with the following modification: the amount of capture beads and detection reagents as well as sample volumes was scaled down five-fold. Samples were acquired on a BD LSR II flow cytometer (BD Biosciences) and data analysed with FCAP Array v3.0 (SoftFlow Inc.).

For intracellular cytokine analyses, cells were suspended in R10 buffer, seeded at 5 x 10^6^ cells/ml in 5 ml polystyrene round-bottom tubes (BD Falcon), and incubated with 250 ng/ml phorbol 12-myristate 13-acetate (PMA; Sigma-Aldrich), 500 ng/ml ionomycin (Sigma-Aldrich) and 10 μg/ml brefeldin A (Sigma-Aldrich), or brefeldin A alone as a negative control, for 3 hours at 37°C, 5% CO_2_. Cells were then washed with R10 buffer followed by staining for flow cytometry analysis.

### Flow Cytometry

Cells were fluorescently labelled for 30–60 minutes on ice with the following antibodies and reagents: BV510-conjugated rat anti-mouse CD45 (clone 30-F11; BioLegend), BV605-conjugated Armenian hamster anti-mouse TCRβ (H57-597; BD Biosciences), PE-conjugated rat anti-mouse IL-10 (JES5-16E3; eBioscience), PE-CF594-conjugated rat anti-mouse CD4 (RM4-5; BD Biosciences), PerCP-Cy5.5-conjugated mouse anti-human/mouse T-bet (4B10; eBioscience), PE-Cy7-conjugated rat anti-mouse IFN-γ (XMG1.2; BioLegend), APC-conjugated rat anti-mouse/rat FoxP3 (FJK-16s; eBioscience), AF700-conjugated rat anti-mouse CD19 (6D5; BioLegend), AF700-conjugated rat anti-human/mouse B220 (RA3-6B2; eBioscience), AF700-conjugated mouse anti-mouse NK1.1 (PK136; BioLegend), AF700-conjugated rat anti-mouse Ter-119 (TER-119; BioLegend), biotin-conjugated rat anti-mouse CD8α (53–6.7; BioLegend), APC-eF780-conjugated streptavidin (eBioscience), along with Violet Live/Dead (Life Technologies) according to manufacturer’s instructions to label dead cells. Cells were stained intracellularly with FoxP3/Transcription factor staining buffer set (eBioscience) according to manufacturer’s instructions. Cells were analysed on a BD LSR II flow cytometer, and data analysed with FlowJo software v9.7 (Tree Star Inc.). Dead cells and aggregates were excluded from all analyses.

### Amplicon Sequencing

Bacterial DNA from caecal and faecal samples were extracted using a NucleoSpin soil kit (Macherey-Nagel) according to manufacturer’s instructions. DNA yield and integrity were assessed using a Nanodrop and agarose gel electrophoresis, respectively. The PCR-based library formation was performed using 10 ng bacterial DNA, 0.2 μM of each barcoded forward and reverse primer, 0.2 mM dNTPs and 0.5 units Phusion high fidelity DNA polymerase (Thermo Scientific) in a total volume of 25 μl. To target the 16S rRNA gene’s variable region 4 (V4) a forward primer 515F (5’ AATGATACGGCGACCACCGAGATCTACAC NNNNNNNN TATGGTAATTGTGTGCCAGCMGCCGCGGTAA 3’; “N” indicates the nucleotides of the barcode sequence) and a reverse primer 806R (5’ CAAGCAGAAGACGGCATACGAGAT NNNNNNNNNNNN AGTCAGTCAG CC GGACTACHVGGGTWTCTAAT 3’) were used, both with Illumina adaptor sequences in the 5’ end [41, 42]. Cycling condition was as follows: 98°C for 30 seconds followed by 35 cycles of 98°C for 5 s, 56°C for 20 s and 70°C for 20 s. PCR products were purified and normalised to 1–2 ng/μl using the SequalPrep Normalisation Plate kit (Life Technologies Europe). Subsequently, samples were pooled (2 μl of each sample) and quantified using a KAPA Library Quantification Kit (KAPA Biosystems) on a Stratagene Mx3000 (Agilent Technologies Denmark). 6.65 pM library and 0.35 pM PhiX Control v3 (Illumina) was sequenced using an Illumina MiSeq V2 PE500 cartridge (500 cycles) on an Illumina MiSeq.

### Bioinformatics

Generated sequences were analysed using qiime_pipe (https://github.com/maasha/qiime_pipe) using QIIME v1.7.0 with default settings, which performs quality-based sequence trimming, primer removal and assembly of paired-end sequences followed by the execution of a QIIME workflow including chimera checking[41]. De novo OTU-picking was performed using UCLUST[43] with 97% sequence similarity. Representative sequences were assigned taxonomy against the Greengenes database v11_2[44] using the RDP-classifier[45] with an 80% confidence threshold. Subsequent analyses were performed in R v3.1.1 using the metagenomeSeq[46], PhyloSeq[47], Vegan[48] and GGplot2[49] packages. Data was filtered for low-abundance OTUs by removal of OTUs present in fewer than 3 of the 140 samples and with a relative abundance across all samples ≤0.005%. A single day 35 faecal sample from one of the infected mice and a single day 0 caecal sample from one of the uninfected mice were left out due to our cut off of at least 10,000 sequences per sample after filtering. Analyses in R were performed with an average of 25,060 ± 6,480 (SD) sequences per sample before, and with 20,912 ± 5,091 (SD) sequences per sample after filtering. Alpha and gamma diversity were estimated using unfiltered data. Beta diversity was performed using filtered data and calculated for time point/treatment using Vegan. Read counts were normalised with metagenomeSeq[46] that uses a cumulative-sum scaling in which raw counts are divided by the cumulative sum of counts up to a particular quantile. Statistical analyses for Table 1 and S2 Table comparing uninfected with infected were performed on data filtered based on effective sample sizes. Taxa were not included if they had fewer than X effective number of positive samples, where X is the median of estimated effective samples per feature calculated using metagenomeSeq. Phylogenetic analyses were conducted using 16S rRNA gene sequences from the given family downloaded from The Ribosomal Database Project (RDP)[50]. The representative sequences, from the OTUs classified to be members of the given family were combined with the RDP sequences. Sequence-alignment using MUSCLE[51] and phylogenetic tree building using the Maximum Likelihood method based on the Tamura-Nei model[52] were performed using MEGA v6.06[50]. All sequence data is available from the European Nucleotide Archive (ENA) with study accession number: PRJEB6560. Immunological raw data is available from Dryad Digital Repository (doi:10.5061/dryad.md0vg).

**Table 1:** Bacterial taxa that differed significantly within faecal samples after *T*. *muris* infection.

						Prevalence		Counts		Abundance (%)				
Time after infection	Phylum	Class	Order	Family	Genus	Uninfected	Infected	Uninfected	Infected	Uninfected	SEM	Infected	SEM	Adj. P-value
**13 days**	Actinobacteria	Actinobacteria	Bifidobacteriaceae	Bifidobacteriaceae	**Bifidobacteriumᛏ**	8/10	10/10	1674	2663	0.66	0.36	0.88	0.56	4.6E-02
**20 days**	Firmicutes	**Bacilliᛏ**				10/10	10/10	3334	11805	1.65	0.24	4.49	0.83	4.1E-02
			**Lactobacillalesᛏ**			10/10	10/10	3325	11784	1.64	0.24	4.48	0.83	3.9E-02
				**Lactobacillaceaeᛏ**		10/10	10/10	3316	11676	1.64	0.24	4.44	0.83	4.0E-02
					**Lactobacillusᛏ**	10/10	10/10	3303	11638	1.63	0.24	4.43	0.83	3.1E-02
	Proteobacteria	**Deltaproteobacteriaᛎ**				10/10	10/10	1820	1020	0.89	0.12	0.40	0.06	3.4E-02
			**Desulfovibrionalesᛎ**			10/10	10/10	1820	1020	0.89	0.12	0.40	0.06	4.4E-02
				**Desulfovibrionaceaeᛎ**		10/10	10/10	1803	1008	0.88	0.11	0.40	0.06	4.0E-02
	Bacteroidetes	Bacteroidia	Bacteriodales	**Rikenellaceaeᛏ**		10/10	10/10	2845	8318	1.45	0.20	3.15	0.55	2.7E-02
					**Alistipesᛏ**	10/10	10/10	2832	8280	1.45	0.20	3.13	0.55	2.0E-02
				Porphyromonadaceae	**Parabacteroidesᛎ**	9/10	7/10	95	30	0.05	0.01	0.01	0.00	4.7E-02
					**Odirobacterᛏ**	10/10	10/10	3218	11353	1.61	0.31	4.35	0.98	2.2E-02
**27 days**	Firmicutes	**Erysipelotrichiᛏ**				10/10	10/10	9197	11665	4.78	1.84	5.10	3.48	4.0E-03
		**Bacilliᛏ**				10/10	10/10	2840	28576	1.32	0.16	11.41	3.12	4.0E-03
			**Lactobacillalesᛏ**			10/10	10/10	2793	28565	1.30	0.15	11.40	3.12	6.8E-03
				**Lactobacillaceaeᛏ**		10/10	10/10	2790	28543	1.30	0.15	11.39	3.12	2.0E-04
					**Lactobacillusᛏ**	10/10	10/10	2790	28433	1.30	0.15	11.35	3.11	7.0E-04
		Clostridia	Clostridiales	**Ruminococcaceaeᛎ**		10/10	10/10	5769	4295	2.57	0.30	1.78	0.46	1.7E-02
	Bacteroidetes	Becteroidia	Bacteroidales	Porphyromonadaceae	**Barnesiellaᛎ**	10/10	10/10	2865	1416	1.39	0.20	0.63	0.19	4.0E-02
**35 days**	Firmicutes	**Bacilliᛏ**				10/10	9/9	1801	29309	0.94	0.25	14.84	3.41	6.6E-05
			**Lactobacillalesᛏ**			10/10	9/9	1795	29297	0.93	0.25	14.84	3.41	3.5E-04
				**Lactobacillaceaeᛏ**		10/10	9/9	1785	29123	0.93	0.24	14.74	3.38	6.1E-04
					**Lactobacillusᛏ**	10/10	9/9	1782	28935	0.93	0.24	14.65	3.36	2.1E-05
		**Erysipelotrichiᛎ**				10/10	9/9	10924	5944	5.62	1.38	2.92	1.73	6.6E-05
			**Erysipelotrichalesᛎ**			10/10	9/9	10924	5944	5.62	1.38	2.92	1.73	3.5E-04
				**Erysipelotrichaceaeᛎ**		10/10	9/9	10924	5944	5.62	1.38	2.92	1.73	8.4E-05
					**Allobaculumᛎ**	10/10	9/9	10804	5892	5.56	1.38	2.90	1.72	2.8E-03
		Clostridia	Clostridiales	Lachnospiraceae	**Roseburiaᛎ**	10/10	6/9	163	16	0.08	0.03	0.01	0.00	1.5E-02
				Ruminococcaceae	**Oscillibacterᛏ**	10/10	8/9	1611	526	0.85	0.16	0.25	0.08	1.5E-02
					**Butyricicoccusᛏ**	10/10	7/9	55	131	0.03	0.01	0.06	0.03	2.5E-02
	Proteobacteria	Burkholderiales	Burkholderiales	Alcaligenaceae	**Parasutterellaᛏ**	10/10	9/9	1440	9032	0.71	0.16	4.30	1.75	1.6E-02
		Alphaproteobacteria	Sphingomonadales	Sphingomonadaceae	**Sphingomonasᛏ**	8/10	7/9	24	54	0.01	0.00	0.03	0.01	9.7E-03
	Gammaproteobacteria	Gammaproteobacteria	Oceanospirillales	Halomonadaceae	**Halomonasᛏ**	10/10	7/9	22	32	0.01	0.00	0.02	0.00	2.5E-02
	Bacteroidetes	Becteroidia	Bacteroidales	Porphyromonadaceae	**Barnesiellaᛎ**	10/10	7/9	3437	1319	1.74	0.27	0.69	0.38	8.8E-03

List of significantly (adj. p-value <0.05) increased or decreased abundance of bacteria at multiple taxonomic ranks when comparing infected with uninfected samples at the given time points. Data are illustrated with the prevalence for each given bacteria, absolute counts, and relative abundance. Significantly increased or decreased bacterial groups are depicted in bold letters with up- or down arrows, respectively. Statistics were performed using metagenomeSeq[46].

## Results

### Chronic *T*. *muris* Infection Induces Major Changes in the Gut Microbiota

To examine whether chronic *T*. *muris* infection affects the composition of the intestinal microbiota, we infected male C57BL/6 mice with a low dose of *T*. *muris* eggs by oral gavage, and collected faecal samples at various time points after infection, coinciding with the different larval developmental stages (S1 Fig). Five weeks after infection, the experiment was terminated and the caecum and colon contents were collected. To confirm the chronic nature of the infection, we assessed the intestinal worm burden of a parallel group of mice infected at the same time. As expected, none of the mice had cleared the infection after five weeks, harbouring 20 ± 3 worms (mean ± SD; n = 5) and were thus, by definition, chronically infected.

**Fig 1:**
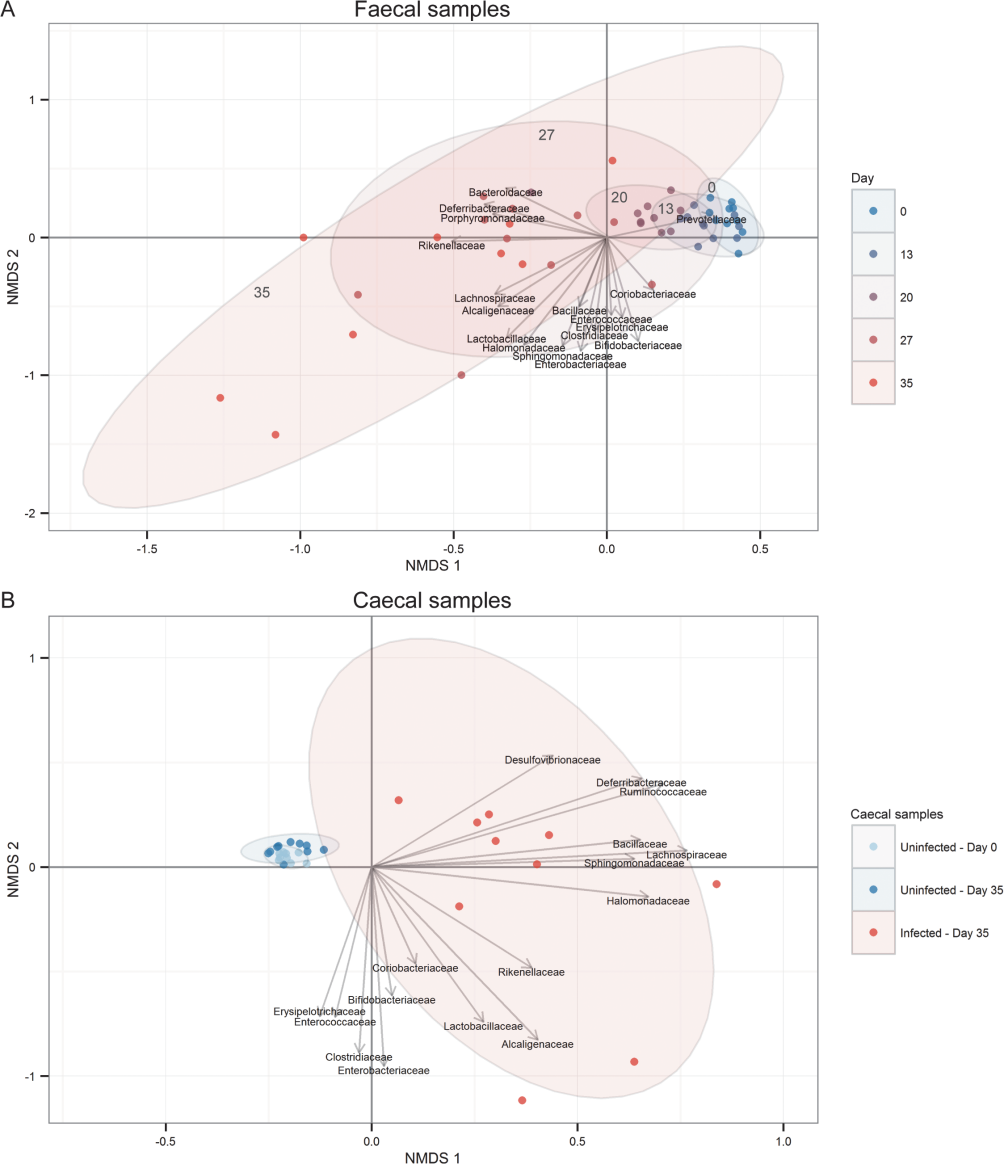
Time-dependent changes of the microbiota diversity due to chronic *T*. *muris* infection. Non-metric Multi-Dimensional Scaling (NMDS) plot using Bray-Curtis dissimilarity indices from (A) faecal microbiota from 10 infected mice sampled at various time points from day 0 to day 35, and (B) caecal microbiota from uninfected mice at day 0 and uninfected/infected mice at day 35. Ellipses are labelled according to the corresponding day of analysis. Relative abundance at family level was fitted as vectors based on 9999 permutations and scaled by their correlation coefficient.

Next, we investigated the composition of the intestinal microbiota utilising 16S rRNA gene-based sequencing and found that chronic *T*. *muris*-infection induced clear changes in microbial communities of the large intestine (Fig 1). While minor alterations were seen at early time points after infection, the first substantial microbial changes appeared from day 20 (Fig 1 and S2 Fig and S1 Table for statistical summary). The changes became more pronounced with time (Fig 1 and S2 Fig and S3 Fig) and were very distinct at day 35 after infection, compared to the uninfected mice (Fig 1 and S2 Fig and S1 Table for statistical summary). The infection had similar effects on the faecal and caecal microbiota (S4 Fig). Importantly, we detected only very minor changes over time in the microbial composition of the uninfected mice (S5 Fig), demonstrating that differences observed in the infected mice reflected infection-dependent changes. Thus, chronic infection with *T*. *muris* is associated with pronounced changes in the microbiota.

### Chronic *T*. *muris* Infection Decreases Alpha and Gamma, but Increases Beta Diversity of the Microbiota

**Fig 2:**
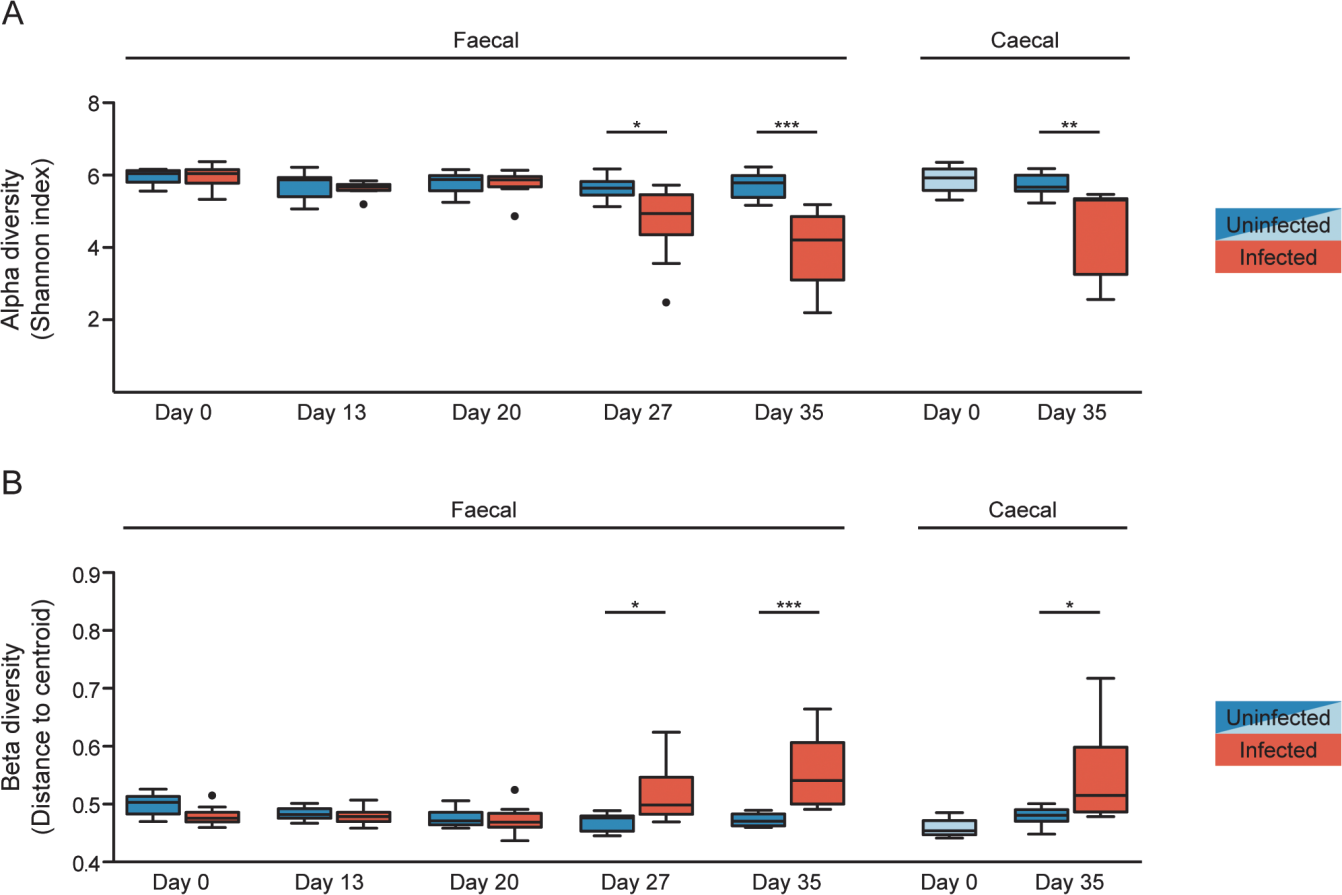
Chronic *T*. *muris* infection results in decreased alpha but increased beta diversity of the microbiota. (A) Median alpha diversity based on Shannon index of unfiltered microbiota data for faecal and caecal samples. The upper and lower whiskers correspond to the 25^th^ and 75^th^ percentiles. (B) Median beta diversity based on Sørensen index for faecal and caecal samples. The whiskers correspond to the 25^th^ and 75^th^ percentiles. Statistical analyses were performed with one-way ANOVA, followed by Tukey’s post-test for multiple comparisons using Prism (GraphPad software). The light blue colour for uninfected caecal samples indicates the ten mice sacrificed at day 0, and therefore not repeated sampling as for the faecal samples. The following definitions were used to denote statistical significance: * (p<0.05), ** (p<0.01), *** (p<0.001), while p>0.05 was considered not significant (NS).

In order to examine whether chronic *T*. *muris* infection influenced the diversity of the intestinal microbiota we determined alpha (within sample) and beta (between samples) diversity for each sample and group, respectively. Alpha diversity analysis was performed on unfiltered data using Shannon index (Fig 2A). The alpha diversity of the faecal samples from the infected mice was significantly decreased after 27 days of infection, and even further decreased after 35 days, while no decrease was observed in the uninfected mice. Similarly, a significant decrease in the alpha diversity in caecal samples was evident after 35 days of infection (Fig 2A). By contrast, beta diversity between the faecal samples from the infected mice was increased after 27 and 35 days, and similarly, increased beta diversity was observed in caecal samples after 35 days (Fig 2B). We therefore investigated whether there was an overall gain or loss of diversity by examining the combined microbiota from all infected mice (gamma diversity) compared to all the uninfected mice. Thus, all untrimmed data were pooled according to treatment and the gamma diversity (between groups) of the infected and uninfected pools was compared using the Shannon index. In accordance with the alpha diversity, we found a decline in gamma diversity at day 27 and 35 after infection in the faecal samples and at day 35 for the caecal samples (S6 Fig). Together, these data demonstrate that chronic *T*. *muris* infection causes an overall decrease in microbial diversity of the large intestine.

### *T*. *muris* Infection Alters the Composition of the Faecal and Caecal Microbiota

Next, we examined the alterations in intestinal microbial composition following *T*. *muris* infection by taking taxonomical classifications of operational taxonomic units (OTU) into account. At the phylum level, *T*. *muris* infection led to increased abundance of *Firmicutes* (day 27 and 35) and *Proteobacteria* (day 35) while decreasing *Bacteroidetes* (day 27 and 35) in the faecal samples (p<0.05, repeated-measures ANOVA, S7 Fig). *Firmicutes*, *Proteobacteria* and *Bacteroidetes* accounted for more than 90% of the microbiota at all time points. The *T*. *muris* infection increased the relative abundance of *Firmicutes* in faecal samples from 37 ± 3% to 43 ± 6% between day 0 and 35. Likewise, the caecal samples from the infected mice contained 58 ± 6% *Firmicutes* at day 35 compared to 30 ± 2% and 36 ± 2% in the uninfected mice sacrificed at day 0 and 35, respectively. *Proteobacteria* and *Bacteroidetes* are traditionally lipopolysaccharide-containing gram-negative bacteria whereas *Firmicutes* are, with minor exceptions, gram-positive bacteria, thus, the microbiota was characterised by an increased proportion of gram-positive bacteria after infection due to the increase in *Firmicutes*.

**Fig 3:**
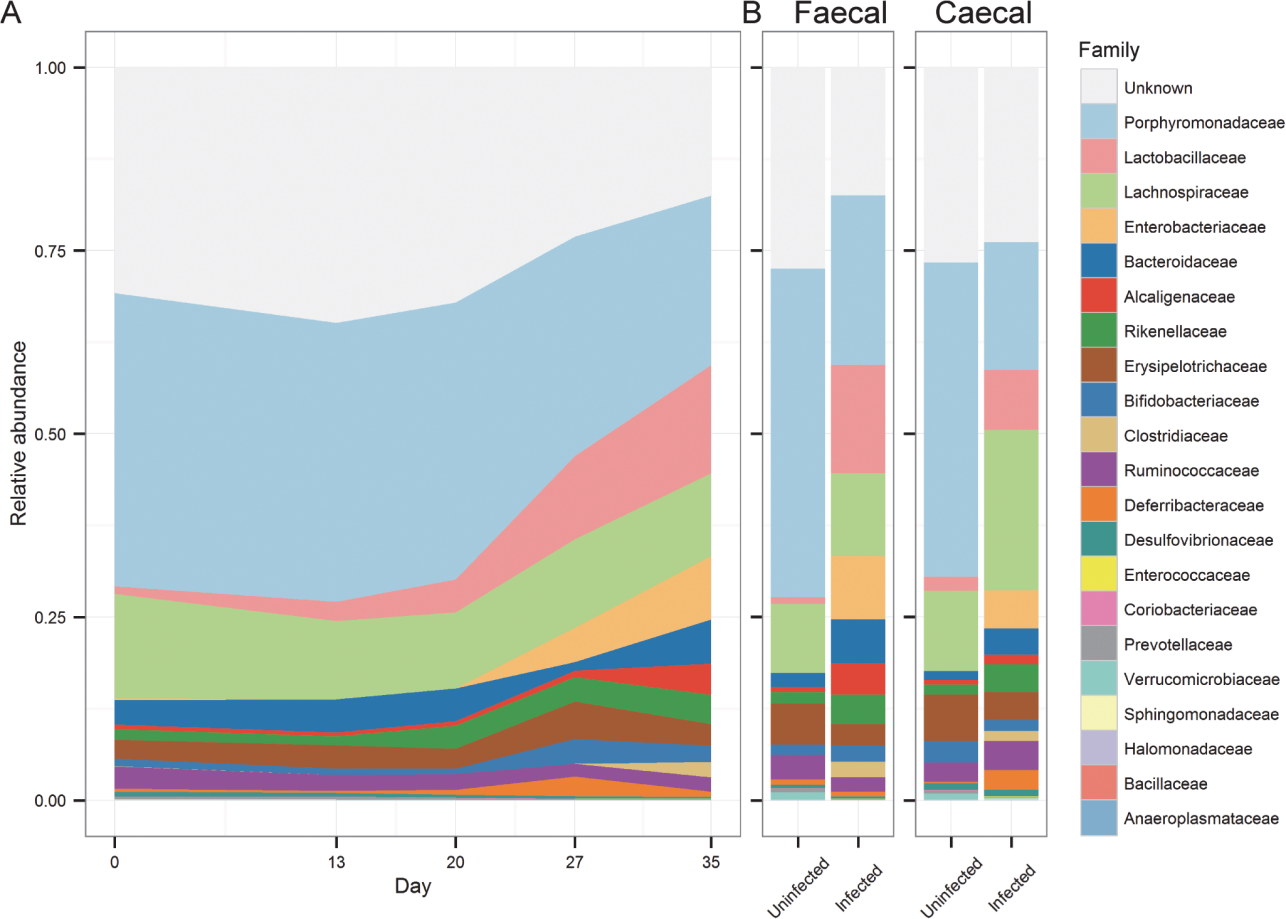
Chronic *T*. *muris* infection affects the composition of the microbiota. Taxa summary plots at family level showing (A) changes in microbiota composition of faecal samples from the infected mice from day 0 to day 35, and (B) the microbiota composition of uninfected and infected mice at day 35 for faecal and caecal samples. “Unknown” refers to OTUs that we were unable to classify. Data represent mean relative abundance.

**Fig 4:**
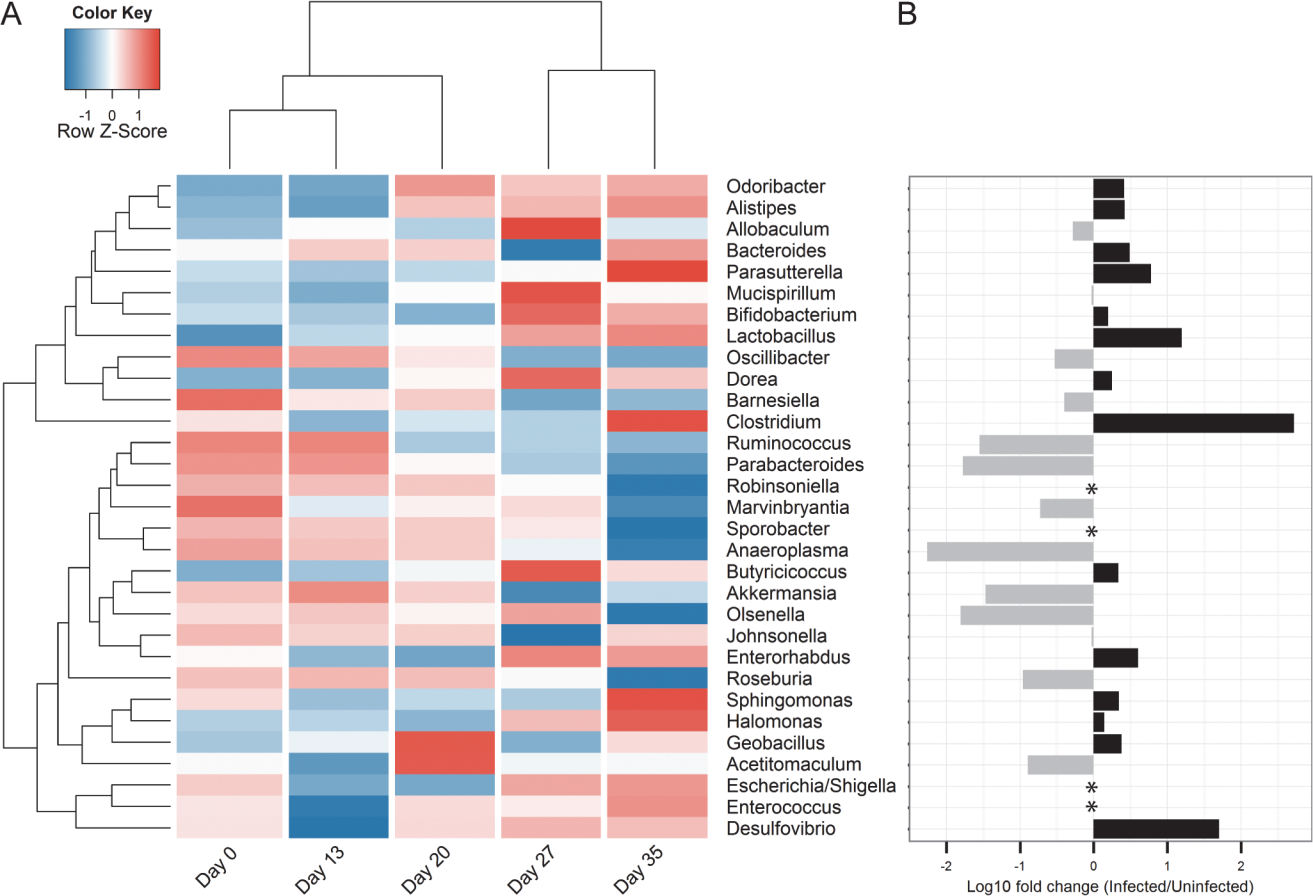
Chronic *T*. *muris* infection alters the relative abundance of multiple genera. (A) Heat-map illustrating changes over time in mean relative abundance at genus level for faecal samples from infected mice. Data are log_10_ transformed and colour-scaled in the horizontal direction. Blue indicates low values and red indicates high values. Dendrograms are based on hierarchical cluster analysis with Bray-Curtis dissimilarity indices. (B) Log_10_ fold change between infected and uninfected faecal samples from day 35. (*) Indicates that the genus was undetected in either infected or uninfected samples. Detected only in uninfected: *Robinsoniella* (0.003%), *Sporobacter* (0.06%). Detected only in infected: *Escherichia/Shigella* (0.06%), *Enterococcus* (0.04%).

To further investigate the changes in the microbiota composition following the *T*. *muris* infection, we analysed the microbiota composition in a taxa summary plot on family level (Fig 3), statistical analysis on multiple taxonomic ranks (Table 1), heat-mapping at genus level, including hierarchical cluster analysis (Fig 4), and phylogenetic tree analysis to identify candidates at species level (S8 Fig). Hierarchical cluster analysis of Bray-Curtis dissimilarity indices identified day 0 and day 13 to be the most similar, with day 20 as the closest relative, while day 27 and 35 were distinctly clustered on two separated branches (Fig 4), indicating that major changes in the microbiota occurred from day 20 after the infection with *T*. *muris*.

Using metagenomeSeq [46] we identified bacteria that were significantly altered due to infection at any given time point. We focused on the most common effects of the infection, which we defined as affected taxa detected in at least as many or more samples compared to the median of the estimated effective sample size, as calculated by MetagenomeSeq. With an increase at day 13 after infection, *Bifidobacterium* was identified as the only significantly affected genus before the major changes to the microbiota occurring at day 20 and onward (Table 1). The most notable change observed was an increase in the relative abundance of the bacterial family *Lactobacillaceae* that remained relatively unaltered until day 20, after which it gradually increased in abundance from <5% between day 0 and 20, to 11% and 15% after days 27 and 35, respectively (Table 1 and Fig 3). While the increase in *Lactobacillaceae* occurred in 8 out of 9 mice, other changes were less general (S9 Fig), further illustrating the variability between individual mice as reflected by the increase in beta diversity described earlier. Interestingly, some genera were affected only at certain time points after infection and then remained stable (Fig 4 and Table 1). Among these we detected an increase in the relative abundance of *Alistipes* and *Odoribacter* from the *Bacteroidales* family at day 20, as well as a decrease in *Allobaculum* and *Barnesiella* and a sharp increase in *Parasutterella* at day 35 (Fig 4 and Table 1). The abundance of *Lactobacillus*, *Allobaculum* and *Barnesiella* were also found to be significantly affected in the caecal samples by the infection with the addition of a >10-fold increased abundance of *Mucispirillum* and a decrease of the low-abundant *Sporobacter* selectively in the caecal samples (S2 Table).

By phylogenetic tree analysis, comparing the representative sequences for the OTUs classified within each family and 16S rRNA gene sequences downloaded from the Ribosomal Database Project[50], we identified candidate species for the *Lactobacillaceae* family affected by *T*. *muris* infection. A single OTU (L#1 in S8 Fig) accounted for more than 80% of the relative abundance in faecal samples 35 days after infection, the sequence of which was identical to that of *L*. *apodemi*, *L*. *murinus* and *L*. *animalis* (S8 Fig). The second most abundant OTU was identical to *L*. *gasseri* and *L*. *taiwanensis*. The same OTUs and similar relative abundance within families were found for caecal samples (data not shown).

### A Skewed Intestinal Regulatory/Inflammatory T Cell Balance is Induced upon Chronic *T*. *muris* Infection

Finally, we wanted to investigate whether the profound changes observed in the microbiota composition correlated with infection-driven responses in the intestinal immune compartment, which could indicate a potential causal link between these processes. CD4^+^ T cells are crucial effector cells during acute *T*. *muris* infections. At low doses of infection both CD4^+^ and CD8^+^ T cells polarise into IFN-γ-secreting effector cells, resulting in the host being unable to expel the worms[53]. In order to confirm that the low infection dose resulted in induction of a Th1 response we therefore tracked and profiled the accumulation of adaptive immune cells in the large-intestinal lamina propria (LI LP) and draining mesenteric lymph nodes (MLN) following chronic *T*. *muris* infection. Initial experimentation indicated that there were substantial changes to the intestinal immune system at day 35 after infection, leading us to conduct an additional experiment, with analysis at key time points concurrent with the kinetics of the observed alterations in the microbiota. Moreover given the established capacity of certain bacterial species[54–56], as well as some parasites[57, 58], to promote induction of regulatory T cells (Treg), we also assessed the generation of these cells during chronic *T*. *muris* infection.

**Fig 5:**
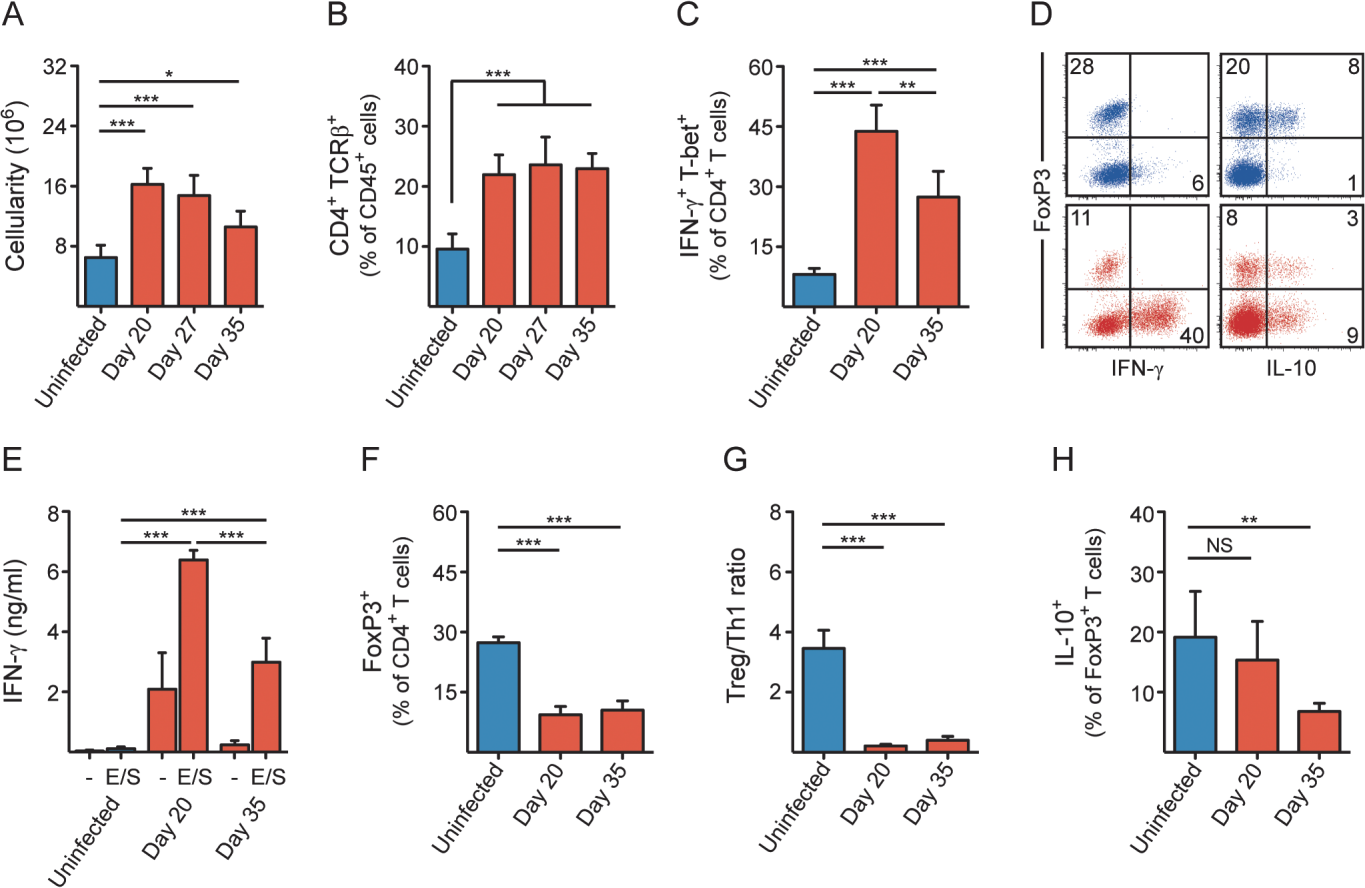
Chronic *T*. *muris* infection alters the regulatory/inflammatory T cell balance in the large intestine. (A) Haematopoietic cell numbers (cellularity) in the LI LP of *T*. *muris*-infected and uninfected mice. (B-C) Proportion of (B) CD4^+^ TCRβ^+^ cells, and (C) IFN-γ^+^ T-bet^+^ CD4^+^ T cells in the LI LP of *T*. *muris*-infected and uninfected mice. (D) Representative flow cytometry plots of FoxP3, IFN-γ and IL-10-expressing CD4^+^ T cells in the LI LP. Numbers indicate frequencies of CD4^+^ T cells. Blue = uninfected, red = *T*. *muris*-infected (day 20). (E) *T*. *muris*-derived E/S antigen-specific secretion of IFN-γ by cells isolated from the LI LP of *T*. *muris*-infected and uninfected mice after *ex vivo* stimulation for 48 h. (F) Proportion of FoxP3^+^ CD4^+^ T cells in the LI LP of *T*. *muris*-infected and uninfected mice. (G) Ratio between FoxP3^+^ and IFN-γ^+^ T-bet^+^ CD4^+^ T cells in the LI LP of *T*. *muris*-infected and uninfected mice. (H) Proportion of IL-10^+^ FoxP3^+^ CD4^+^ T cells in the LI LP of *T*. *muris*-infected and uninfected mice. Bar graphs are displayed as mean (n = 6) with standard deviation. Statistical analyses were performed with one-way ANOVA, followed by Tukey’s post-test for multiple comparisons using Prism (GraphPad software). The following definitions were used to denote statistical significance: * (p<0.05), ** (p<0.01), *** (p<0.001), while p>0.05 was considered not significant (NS).

As expected, mice chronically infected with *T*. *muris* developed intestinal inflammation with an accumulation of haematopoietic cells, both in the LI LP (Fig 5A) and MLN (S10A Fig). This was apparent at day 20 and persisted throughout the course of infection. Similarly, the proportion of CD4^+^ T cells in the LI LP was also increased at day 20 and remained stable with time (Fig 5B), with a similar trend for CD8^+^ T cells (S10B Fig). In order to investigate potential driving forces from the microbiota on the nature of the adaptive immune response after infection, we focused our analysis on time points either prior to (at day 20) or after (at day 35) the major alterations observed in microbial communities, most notably the *Lactobacillaceae*. Consistent with our expectations, a large fraction of the total CD4^+^ T cells in the LI LP expressed classical markers of inflammatory Th1 cells; the cytokine IFN-γ (Fig 5C and 5D) and the transcriptional regulator T-bet (S10C Fig) after infection. The Th1 response was established early after infection (at latest day 20) but interestingly abated as *T*. *muris* reached adulthood (Fig 5C). CD8^+^ T cells, although fewer in numbers relative to CD4^+^ T cells, were also positive for IFN-γ and T-bet to a similar extent (S10D Fig). Consistent with the T cell response, single-cell suspensions of both LI LP (Fig 5E) and MLN (S10E Fig) from infected mice showed a dramatic IFN-γ secretion upon stimulation with *T*. *muris*-derived excretory/secretory (E/S) antigens. Moreover, we detected a slight induction of FoxP3^+^ Tregs in the MLN as the infection progressed (S10F Fig). However, contrary to what has been reported in other parasite models, we did not detect an increased proportion of FoxP3^+^ CD4+ T cells in the LI LP at this infection dose, but rather a substantial decrease (Figs 5D and 5F), a change that was apparent already at day 20 and remained as the infection progressed (Fig 5F). Strikingly, the ratio between regulatory and inflammatory CD4^+^ T cells was reduced almost 16-fold in the LI LP as a consequence of infection (Fig 5G). Treg cells were not only fewer in proportion, but also seemed less prone to produce interleukin-10 (IL-10) at day 35 (Fig 5H and S10C Fig), a cytokine known to be involved in tolerogenic and anti-inflammatory responses. In fact, most of the T cell-derived IL-10 during infection was produced by FoxP3^-^ IFN-γ^+^ cells (Fig 5D and S10G Fig), and was reflected by IL-10 secretion from E/S-stimulated LI LP cells (S10H Fig). Taken together, these results demonstrate that low dose infection with *T*. *muris* led to chronic inflammation of the large intestine, with a decreased ratio between regulatory and inflammatory CD4^+^ T cells, which was clearly manifested after 20 days of infection and remained as the infection progressed.

## Discussion

We have performed a comprehensive study analysing the influence of chronic *T*. *muris* infection on the murine gut microbiota using 16S rRNA gene-based sequencing. By infecting mice with *T*. *muris*, we have been able to longitudinally study the effect of chronic nematode infection in a highly controlled manner, with sampling at multiple time points. We found that persistent worm infection led to a decrease in bacterial diversity of the large-intestinal microbiota as compared to uninfected mice, with an associated increase in the relative abundance of *Lactobacillaceae*. In parallel, we detected an overall change in the intestinal regulatory/inflammatory T cell balance following chronic *T*. *muris* infection.

Two recent publications describe the effect of *T*. *trichiura* infection on the intestinal microbiota of infected humans[38, 39]. Compared with the effects of *T*. *muris* infection reported here, the effect of *T*. *trichiura* infection in humans appeared less drastic. Cooper *et al*. found no decrease in the alpha diversity in *T*. *trichiura*-infected children compared to an uninfected control group, and similarly no apparent difference was detected following curative treatment[38]. In contrast, Lee *et al*. detected a minor increase in alpha diversity of the intestinal microbiota in infected individuals, which included combinations of *T*. *trichiura*, *Ascaris lumbricoides* and hookworm co-infections[39]. However apart from likely species-related differences, human field studies are often less controlled compared to laboratory-based studies in mice (e.g. variation in infection dose, duration and timing of infection), and many additional environmental factors will undoubtedly influence the results, e.g. lifestyle, gender, age, hygiene and/or previous pathogen exposure.

We found that chronic *T*. *muris* infection had a strong effect on the alpha, beta and gamma diversity of both the faecal and caecal microbiota. The decreased alpha diversity suggests that the microbiota became less diverse within each individual mouse, whereas the concurrent increase in beta diversity illustrates that each individual mouse responded differently to infection, resulting in a larger diversity between the mice. Nevertheless, overall we found the gamma diversity to be decreased, indicating that the total number of different species of bacteria existing in an infected population of mice is reduced compared to an uninfected population. Besides parasite infection, a reduced microbial diversity has been observed in patients suffering from IBD[59], indicating that this phenomenon may be a by-product of the inflammatory process itself rather than being a specific outcome of the parasite infection. On the other hand, *Toxoplasma gondii* infection was recently found to induce antigen- as well as commensal-specific T cell responses[60]; it is therefore conceivable that *T*. *muris* infection *per se* also results in bacteria-specific responses, which may be driving at least some of the observed changes.

It remains to be established what drives the pronounced and significant changes in the composition of the intestinal microbiota, especially those occurring from day 20 after infection. Several possibilities can be envisaged, which may involve direct as well as indirect mechanisms via the immune system. The time point for the major changes observed in microbial composition coincides with the larvae progressing from the L2 to the L3 developmental stage, a transition that is associated with the worms increasing in size and starting to protrude from the syncytial tunnels into the intestinal lumen. Thus, the physical presence of larvae in the intestinal lumen may be associated with a larger influence on the intestinal environment that may alter the intestinal microbiota. Coincidentally, the parasite may influence its microenvironment by secreting effector molecules that affect the microbiota directly or indirectly via stimulation of host immune cells. A large fraction of the E/S components secreted by the parasite during infection is made up of serine proteases that have a documented capacity to degrade the major intestinal mucin, *muc2*[61]. Given that many of the intestinal microbes actively attach to the mucous layer it is plausible that the worms, by affecting the integrity of the mucous layer in order to promote their own survival in the intestine, may indirectly also affect the capacity of mucous-adhering bacteria to remain in their intestinal niche[62]. In fact, consistent with such a mechanism, a recent study showed that immune-driven alterations to goblet cell-mediated mucous production led to reduced bacterial attachment to the mucous during *T*. *trichiura* infection in macaques[63]. Finally, there are indications that the parasite itself may be driving the changes to the microbiota independent of the adaptive immune response. Along these lines, Rausch *et al*. found that mice infected with the hookworm *Heligmosomoides polygyrus* responded with a Th2 response and had altered intestinal microbiota composition[10]. However following infection of IL-4Rα^-/-^ mice, which are compromised in their capacity to generate a Th2 response, they found similar changes in the gut microbiota composition, indicating that the hookworm was able to alter the microbiota independently of the IL-4-regulated adaptive Th2 response[10].

The most notable change of the microbiota following the *T*. *muris* infection was an increased abundance of the gram-positive, facultative anaerobic family *Lactobacillaceae*, suggesting a positive correlation between these bacteria and the nematode infection. This is in agreement with Walk *et al*, who found the abundance of the *Lactobacillus* genus to be increased following infection with *H*. *polygyrus*, and speculated that the increase in lactobacilli could be an immune-modulating effect of *H*. *polygyrus* infection as part of a mutualistic relationship with the resident bacteria[11]. Moreover, Reynolds *et al*. identified the species *L*. *taiwanensis* to be increased by *H*. *polygyrus* infection, and showed that administration of *L*. *taiwanensis* to BALB/c mice facilitated subsequent infection with the nematode[64]. Furthermore, Dea-Ayuela *et al*. found that treating mice orally with either viable or dead *L*. *casei* enhanced the susceptibility of B10Br mice to *T*. *muris* infection[34], and that viable *L*. *casei* abrogated the IFN-γ response in MLN after infection. We found a similarly decreased IFN-γ response in both the LI LP and MLN between day 20 to 35, concurrent with an increase in relative abundance of lactobacilli, indicating a possible negative correlation between the IFN-γ response and the presence of lactobacilli. Taken together, this may indicate that the increase in lactobacilli during infection is a process that favours the survival of *T*. *muris*, and vice versa, lending credence to the mutualism hypothesis.

Despite prior studies having demonstrated that the microbiota can influence the development and function of the host’s immune system, we detected little correlation between the kinetics of microbial changes and the development of the adaptive immune response. Treg cells play a critical role in controlling immune responses toward both pathogens and the gut microbiota[65], as well as preventing autoimmunity[66]. Furthermore, some parasitic infections are known to induce Treg cells that dampen effector responses[57, 58], in part serving as the rationale for therapeutic worm infection as treatment for human diseases. Our data suggests that this does not seem to be the case for chronic *T*. *muris* infection. Rather, the infection drastically reversed the regulatory/inflammatory balance of the intestinal immune system, with the ratio of regulatory to inflammatory T cells going from ∼4:1 in uninfected mice to ∼1:4 following infection. These findings are surprising also given the documented ability of several *Lactobacillus* species to promote the induction or expansion of Treg cells in various tissues, under different conditions, including parasite infection[64, 67–69]. The change in the balance between regulatory/inflammatory cells was established at an early time point after infection, prior to the observed increase in *Lactobacillaceae*, and remained relatively stable over time, leading us to conclude that other mechanisms control the differentiation and development of the adaptive immune response. We cannot however rule out that the altered microbiota may influence on the immune response at later stages of the infection.

The Treg cells in our experiments appeared less prone to produce IL-10 as the infection progressed. Nonetheless, we did observe an early burst in IL-10 production by FoxP3^-^ IFN-γ^+^ cells, possibly as a compensatory mechanism. However, this also abated with time, as did production of IFN-γ^+^ and the inflammatory response in general. It is therefore possible that *T*. *muris* causes immune exhaustion at later stages of chronic infection. Together, this implies a strategy adopted by the immune system to prevent pathology rather than a direct mechanism by the worm to prevent expulsion, as expulsion cannot take place at these low doses without immune intervention, such as blockade of IFN-γ. It is important to note however that these findings only apply to low dose infections. High dose infections are cleared within 2–3 weeks in most mouse strains [70], and it is possible that Treg cell induction occurs by a different kinetic and plays a different role under these conditions.

In summary, our data demonstrate that chronic infection with the nematode *T*. *muris* results in an altered intestinal microbiota as well as a perturbed immune regulatory/inflammatory balance. Our results are largely consistent and complementary to those of Houlden *et al*. published simultaneously to this paper[71], highlighting the reliability of our findings. These studies are of fundamental importance and considerable relevance, especially given the high incidence of worm infections worldwide and current efforts to use parasitic nematodes to ameliorate disorders associated with perturbations in the immune system.

## Supporting Information

S1 Fig Experimental outline of chronic *T*. *muris* infection. Thirty mice were divided into three groups, each consisting of ten individuals. The first group of ten mice was sacrificed at day 0, and caecal and faecal samples were collected as reference. Of the remaining two groups of then mice, one was infected (“Infected”) with approximately twenty infective *T*. *muris* eggs, while the other was left uninfected (“Uninfected”). Fresh faeces were sampled regularly from mice of both groups and the mice were monitored over time until day 35 after infection, when the mice were sacrificed. Caecum and colon contents were collected and analysed for microbiota composition. * These caecal samples are from ten mice sacrificed at day 0 and do therefore not represent repeated sampling as for the faecal samples.
(EPS)

S2 Fig Early grouping visualised when comparing infected with uninfected faecal samples. NMDS plots using Bray-Curtis dissimilarity indices of faecal microbiota samples from 10 chronically infected and 10 uninfected mice sampled from day 0 to day 35. Ellipses are coloured according to treatment (infected/uninfected). Relative abundance at family level are fitted as vectors-based 9999 permutations and scaled by their correlation coefficient.
(EPS)

S3 Fig Early grouping visualised when comparing faecal samples at different time points after infection. NMDS plots using Bray-Curtis dissimilarity indices of faecal microbiota samples from 10 chronically infected mice sampled from day 0 to day 35. Ellipses are labelled according to the corresponding day of analysis. Relative abundance at family level are fitted as vectors-based 9999 permutations and scaled by their correlation coefficient.
(EPS)

S4 Fig Microbiota samples at day 35 after infection illustrate similar effects of chronic *T*. *muris* on both the faecal and caecal microbiota. NMDS plot using Bray-Curtis dissimilarity indices of colonic and caecal microbiota samples from day 35. Mouse ID numbers are indicated with numbers inside the data points. Faecal and caecal samples are indicated with triangle and circle, respectively. Dotted line separates infected from uninfected samples.
(EPS)

S5 Fig No grouping was apparent as effect of time in the uninfected faecal samples. NMDS plot using Bray-Curtis dissimilarity indices of faecal microbiota samples from 10 uninfected mice sampled from day 0 to day 35.
(EPS)

S6 Fig Chronic *T*. *muris* infection results in decreased gamma diversity of the microbiota. Gamma diversity (measurement of overall diversity of pooled data) based on Shannon index of untrimmed microbiota data for faecal and caecal samples. Data were pooled group- and time point-wise. The light blue colour for caecal samples from uninfected mice indicates the ten mice sacrificed at day 0, and therefore not repeatedly sampled as for the faecal samples. No statistics were applied as the samples were pooled and thereby only provide one single value per time point and treatment.
(EPS)

S7 Fig The microbiota composition is highly affected by chronic *T*. *muris* infection at the phylum level. Taxa summary plots at phylum level showing (A) changes in microbiota composition of faecal samples from the infected mice from day 0 to day 35 and (B) the different microbiota composition between uninfected and infected mice at day 35 for faecal and caecal samples. “Unknown” refers to OTUs that we were unable to classify. Data represents mean relative abundance.
(EPS)

S8 Fig Phylogenetic analysis of *Lactobacillaceae* by Maximum Likelihood-method illustrates species candidates affected by chronic *T*. *muris* infection. The bar-plot illustrates the relative abundance of the OTUs classified within the *Lactobacillaceae* family in day 35 faecal samples from infected mice. Phylogenetic analyses were conducted using the representative sequences from the given OTUs (shown in red) and classified 16S rRNA gene sequences from the *Lactobacillaceae* family downloaded from The Ribosomal Database Project (RDP)[50]. The branch labels contain the RDP sequence identifier number, species name and information on the strain from which the sequence was obtained. The tree with the highest log likelihood (-2535,1977) is shown. The tree is drawn to scale, with branch lengths measured in the number of substitutions per site.
(EPS)

S9 Fig Distinct responses to chronic *T*. *muris* infection in the faecal microbiota at the family level. Taxa summary plots at family level showing changes in microbiota composition at day 35 of faecal samples from each individual (A) uninfected and (B) infected mouse. “Unknown” refers to OTUs that we were unable to classify.
(EPS)

S10 Fig Effects of chronic *T*. *muris* infection on inflammatory and regulatory T cell populations in the LI LP and MLN. (A) Haematopoietic cell numbers (cellularity) in the MLN of *T*. *muris*-infected and uninfected mice. (B) Proportion of CD8α^+^ TCRβ^+^ cells in the LI LP of *T*. *muris*-infected and uninfected mice. (C) Histograms of T-bet (left) and IL-10 (right) expression by IFN-γ^+^ and FoxP3^+^ CD4^+^ T cells, respectively, in the LI LP of *T*. *muris*-infected and uninfected mice. Blue = uninfected, red = *T*. *muris*-infected (day 35), grey = staining control. (D) Proportion of IFN-γ^+^ T-bet^+^ CD8α^+^ T cells, in the LI LP of *T*. *muris*-infected and uninfected mice. (E) *T*. *muris*-derived E/S antigen-specific secretion of IFN-γ by cells isolated from the MLN of *T*. *muris*-infected and uninfected mice after *ex vivo* stimulation for 48 h. (F-G) Proportion of (F) FoxP3^+^ CD4^+^ T cells, and (G) IL-10^+^ IFN-γ^+^ FoxP3^-^ CD4^+^ T cells in the MLN and LI LP, respectively of *T*. *muris*-infected and uninfected mice. (H) E/S antigen-specific secretion of IL-10 by cells isolated from the LI LP of *T*. *muris*-infected and uninfected mice after *ex vivo* stimulation for 48 h. Bar graphs are displayed as mean (n = 6) with standard deviation. Statistical analyses were performed with one-way ANOVA, followed by Tukey’s post-test for multiple comparisons, using Prism (GraphPad software). The following definitions were used to denote statistical significance: * (p<0.05), ** (p<0.01), *** (p<0.001), while p>0.05 was considered not significant (NS).
(EPS)

S1 Table Adonis test of significance illustrates a significant effect of chronic *T*. *muris* on the microbiota. Adonis test of significance performed using Bray-Curtis distance matrix. The following definitions were used to denote statistical significance: * (p≤0.05), ** (p≤0.01), *** (p≤0.001), while p>0.05 was considered not significant.
(DOCX)

S2 Table Bacterial taxa that differed significantly within caecal samples after *T*. *muris* infection. List of significantly (adj. p-value <0.05) increased or decreased abundance of bacteria at multiple taxonomic ranks when comparing infected with uninfected samples at the given timepoints. Statistics were performed using metagenomeSeq[46]. Data are given with the prevalence for the given bacteria, absolute counts, and relative abundance.
(EPS)

S3 Table The Representative Sequences for the OTUs classified in the *Lactobacillaceae* family and used for the Phylogenetic analysis to identify species candidates affected by chronic *T*. *muris* infection . The 9 OTUs named L#1 to L#9 were classified in the *Lactobacillaceae* family according to Greengenes database v11_2 [44] using the RDP-classifier [45] with an 80% confidence threshold.
(DOCX)
