# Supplementary material for: Chronic Trichuris muris Infection Decreases Diversity of the Intestinal Microbiota and Concomitantly Increases the Abundance of Lactobacilli
Source: PLoS One. 2015 May 5;10(5):e0125495. doi: 10.1371/journal.pone.0125495 (PMC4420551; doi:10.1371/journal.pone.0125495)
Supplement: S1 Table — Adonis test of significance performed using Bray-Curtis distance matrix. The following definitions were used to denote statistical significance: * (p≤0.05), ** (p≤0.01), *** (p≤0.001), while p>0.05 was considered not significant. (DOCX) [file pone.0125495.s011.docx]

| **Adonis** | **Group/Timepoint** | **R^2** | **Pr(>F)** | **Significance** |
| --- | --- | --- | --- | --- |
| Effect of time within groups | Uninfected (Faecal) | 0.03614 | 0.002 | ** |
|  | Infected (Faecal) | 0.21397 | 0.001 | *** |
| Effect of infection at each timepoint | Day 0 (Faecal) | 0.06093 | 0.132 |  |
|  | Day 13 (Faecal) | 0.05596 | 0.362 |  |
|  | Day 20 (Faecal) | 0.14239 | 0.001 | *** |
|  | Day 27 (Faecal) | 0.29231 | 0.001 | *** |
|  | Day 35 (Faecal) | 0.35212 | 0.001 | *** |
|  | Day 35 (Caecal) | 0.32360 | 0.001 | *** |
| Effect of time within infected group | Day 0 vs 13 (Faecal) | 0.06962 | 0.060 |  |
|  | Day 13 vs 20 (Faecal) | 0.12369 | 0.001 | *** |
|  | Day 20 vs 27 (Faecal) | 0.16144 | 0.001 | *** |
|  | Day 27 vs 35 (Faecal) | 0.11598 | 0.009 | ** |
