# Supplementary material for: Chronic Trichuris muris Infection Decreases Diversity of the Intestinal Microbiota and Concomitantly Increases the Abundance of Lactobacilli
Source: PLoS One. 2015 May 5;10(5):e0125495. doi: 10.1371/journal.pone.0125495 (PMC4420551; doi:10.1371/journal.pone.0125495)
Supplement: S3 Table — . The 9 OTUs named L#1 to L#9 were classified in the Lactobacillaceae family according to Greengenes database v11_2 [44] using the RDP-classifier [45] with an 80% confidence threshold. (DOCX) [file pone.0125495.s013.docx]

| **ID** | **Sequence** |
| --- | --- |
| L#1 | tacGTAGGTGGCAAGCGTTATCCGGATTTATTGGGCGTAAAGGGAACGCAGGCGGTCTTTTAAGTCTGATGTGAAAGCCTTCGGCTTAACCGGAGTAGTGCATTGGAAACTGGGAGACTTGAGTGCAGAAGAGGAGAGTGGAACTCCATGTGTAGCGGTGAAATGCGTAGATATATGGAAGAACACCAGTGGCGAAAGCGGCTCTCTGGTCTGTAACTGACGCTGAGGTTCGAAAGCGTGGGTAGCAAACagg |
| L#2 | tacGTAGGTGGCAAGCGTTATCCGGATTTATTGGGCGTAAAGGGAACGCAGGCGGTCTTTTAAGTCTGATGTGAAAGCCTTCGGCTCAACCGGAGAATTGCATCAGAAACTGTTGAACTTGAGTGCAGAAGAGGAGAGTGGAACTCCATGTGTAGCGGTGGAATGCGTAGATATATGGAAGAACACCAGTGGCGAAAGCGGCTCTCTGGTCTGTAACTGACGCTGAGGTTCGAAAGCGTGGGTAGCAAACagg |
| L#3 | tacGTAGGTGGCAAGCGTTATCCGGATTTATTGGGCGTAAAGGGAACGCAGGCGGTCTTTTAAGTCTGATGTGAAAGCCTTCGGCTTAACCGGAGTAGTGCATTGGAAACTGGGAGACTTGAGTGCAGAAGAGGAGAGTGGAACTCCATGTGTAGCGGTGAAATGCGTAGATATTAGGAGGAACACCAGTGGCGAAGGCGGCTCTCTGGTCTGCAACTGACGCTGAGGCTCGAAAGCATGGGTAGCGAACagg |
| L#4 | tacGGAGGATGCGAGCGTTATCCGGATTTATTGGGCGTAAAGGGAACGCAGGCGGTCTTTTAAGTCTGATGTGAAAGCCTTCGGCTTAACCGGAGTAGTGCATTGGAAACTGGGAGACTTGAGTGCAGAAGAGGAGAGTGGAACTCCATGTGTAGCGGTGGAATGCGTAGATATATGGAAGAACACCAGTGGCGAAGGCGGCTCTCTGGTCTGCAACTGACGCTGAGGCTCGAAAGCATGGGTAGCGAACagg |
| L#5 | tacgggggatgcgagcgttgtccggatttattgggcgtaaagcGAGCGCAGGCGGTTGCTTAGGTCTGATGTGAAAGCCTTCGGCTTAACCGAAGAAGTGCATCGGAAACCGGGCAACTTGAGTGCAGAAGAGGACAGTGGAACTCCATGTGTAGCGGTGGAATGCGTAGATATATGGAAGAACACCAGTGGCGAAGGCGGCTGTCTGGTCTGCAACTGACGCTGAGGCTCGAAAGCATGGGTATCGAAcagg |
| L#6 | tacGTAGGTGGCAAGCGTTATCCGGATTTATTGGGCGTAAAGCGAGCGCAGGCGGTTGCTTAGGTCTGATGTGAAAGCCTTCGGCTTAACCGAAGAAGTGCATCGGAAACCGGGCGACTTGAGTGCAGAAGAGGACAGTGGAACTCCATGTGTAGCGGTGGAATGCGTAGATATATGGAAGAACACCAGTGGCGAAGGCGGCTGTCTGGTCTGCAACTGACGCTGAGGCTCGAAAGCATGGGTAGCGAAcagg |
| L#7 | tacGTAGGTGGCAAGCGTTGTCCGGATTTATTGGGCGTAAAGCGAGTGCAGGCGGTTCAATAAGTCTGATGTGAAAGCCTTCGGCTTAACCGGAGTAGTGCATTGGAAACTGGGAGACTTGAGTGCAGAAGAGGAGAGTGGAACTCCATGTGTAGCGGTGGAATGCGTAGATATATGGAAGAACACCAGTGGCGAAGGCGGCTCTCTGGTCTGCAACTGACGCTGAGGCTCGAAAGCATGGGtagcgaacagg |
| L#8 | tacGTAGGTGGCAAGCGTTGTCCGGATTTATTGGGCGTAAAGCGAGTGCAGGCGGTTCAATAAGTCTGATGTGAAAGCCTTCGGCTCAACCGGAGAATTGCATCAGAAACTGTTGAACTTGAGTGCAGAAGAGGAGAGTGGAACTCCATGTGTAGCGGTGGAATGCGTAGATATATGGAAGAACACCAGTGGCGAAGGCGGCTCTCTGGTCTGCAACTGACGCTGAGGCTCGAAAGCATGGGtagcgaacagg |
| L#9 | tacGTAGGTGGCAAGCGTTGTCCGGATTTATTGGGCGTAAAGCGAGCGCAGGCGGAAAGATAAGTCTGATGTGAAAGCCCCCGGCTTAACCGAGGAATTGCATCGGAAACTGTGTTTCTTGAGTGCAGAAGAGGAGAGTGGAACTCCATGTGTAGCGGTGGAATGCGTAGATATATGGAAGAACACCAGTGGCGAAGGCGGCTCTCTGGTCTGTAACTGACGCTGAGGCTCGAAAGCATGGGTAGCGAACagg |
